# Evaluating the Laboratory Risk Indicator to Differentiate Cellulitis from Necrotizing Fasciitis in the Emergency Department

**DOI:** 10.5811/westjem.2017.3.33607

**Published:** 2017-05-12

**Authors:** Michael M. Neeki, Fanglong Dong, Christine Au, Jake Toy, Nima Khoshab, Carol Lee, Eugene Kwong, Ho Wang Yuen, Jonathan Lee, Arbi Ayvazian, Pamela Lux, Rodney Borger

**Affiliations:** * Arrowhead Regional Medical Center, Department of Emergency Medicine, Colton, California; † Western University of Health Sciences, College of Osteopathic Medicine of the Pacific, Pomona, California; ‡ California University of Science and Medicine, Colton, California

## Abstract

**Introduction:**

Necrotizing fasciitis (NF) is an uncommon but rapidly progressive infection that results in gross morbidity and mortality if not treated in its early stages. The Laboratory Risk Indicator for Necrotizing Fasciitis (LRINEC) score is used to distinguish NF from other soft tissue infections such as cellulitis or abscess. This study analyzed the ability of the LRINEC score to accurately rule out NF in patients who were confirmed to have cellulitis, as well as the capability to differentiate cellulitis from NF.

**Methods:**

This was a 10-year retrospective chart-review study that included emergency department (ED) patients ≥18 years old with a diagnosis of cellulitis or NF. We calculated a LRINEC score ranging from 0–13 for each patient with all pertinent laboratory values. Three categories were developed per the original LRINEC score guidelines denoting NF risk stratification: high risk (LRINEC score ≥8), moderate risk (LRINEC score 6–7), and low risk (LRINEC score ≤5). All cases missing laboratory values were due to the absence of a C-reactive protein (CRP) value. Since the score for a negative or positive CRP value for the LRINEC score was 0 or 4 respectively, a LRINEC score of 0 or 1 without a CRP value would have placed the patient in the “low risk” group and a LRINEC score of 8 or greater without CRP value would have placed the patient in the “high risk” group. These patients missing CRP values were added to these respective groups.

**Results:**

Among the 948 ED patients with cellulitis, more than one-tenth (10.7%, n=102 of 948) were moderate or high risk for NF based on LRINEC score. Of the 135 ED patients with a diagnosis of NF, 22 patients had valid CRP laboratory values and LRINEC scores were calculated. Among the other 113 patients without CRP values, six patients had a LRINEC score ≥ 8, and 19 patients had a LRINEC score ≤ 1. Thus, a total of 47 patients were further classified based on LRINEC score without a CRP value. More than half of the NF group (63.8%, n=30 of 47) had a low risk based on LRINEC ≤5. Moreover, LRINEC appeared to perform better in the diabetes population than in the non-diabetes population.

**Conclusion:**

The LRINEC score may not be an accurate tool for NF risk stratification and differentiation between cellulitis and NF in the ED setting. This decision instrument demonstrated a high false positive rate when determining NF risk stratification in confirmed cases of cellulitis and a high false negative rate in cases of confirmed NF.

## INTRODUCTION

Necrotizing fasciitis (NF) is a rare but life-threatening soft tissue infection characterized by rapidly progressive necrosis of subcutaneous tissues and deep fascia planes, with resulting skin gangrene and severe systemic infection. 1 The median mortality rate for NF is 32.2% but varies throughout the literature from 8.7% to 76%.^2^,^3^ Patients with NF must be promptly and aggressively treated with surgical intervention to reduce morbidity and mortality.^2^,^4^,^5^ Mortality associated with NF that is not treated with surgical debridement approaches 100%, even with antibiotic treatment. 1 The extremities, groin, and abdomen are the sites most frequently affected by the disease. 4

Early diagnosis of NF is difficult due to the low rate of incidence, lack of knowledge of various presentations, and elusive clinical presenting signs and symptoms. 1 The Laboratory Risk Indicator for Necrotizing Fasciitis (LRINEC) score was developed as a diagnostic tool to potentially aid practitioners in early detection of NF. 6 A LRINEC score between 0 and 13 can be calculated based on levels of serum leukocytes, glucose, sodium, C-reactive protein (CRP), creatinine, and hemoglobin. All six components of the LRINEC score are required for valid calculation. LRINEC scores ≥8 fall in the high-risk category, LRINEC scores of 6–7 are moderate risk, while scores ≤5 are considered low risk. 6 Previous evidence suggested that a patient with a LRINEC ≥6 should be further evaluated for NF diagnosis. 6

Validation studies determined the LRINEC score to have low predictive value.^7^,^8^ Burner and colleagues reported that the LRINEC score was not sufficiently sensitive to rule out NF.^9^ Additionally, CRP value is not routinely collected in the emergency department (ED), which presents a barrier for the effective utilization of the LRINEC score as a predictive tool.

Soft tissue infections, including cellulitis and NF, are difficult to differentiate due to similarities at initial onset.^9^,^10^ Pain and progressive erythema are common presenting symptoms of both these infections.^10^,^11^ However, the sequela of NF is far more severe than cellulitis including sepsis, loss of limbs, and death. Further, diabetes is a known risk factor for developing these soft tissue infections. Regarding the LRINEC score, Burner and colleagues reported a higher discrimination ability among the diabetes population in cases of correctly predicted NF.^9^

To our knowledge, no previous studies have assessed the performance of the LRINEC score against confirmed cases of cellulitis. This study further aimed to evaluate the predictive ability of the LRINEC score among confirmed cases of NF. Findings from this study may aid emergency physicians (EP) in better understanding the clinical application of the LRINEC score and its accuracy as a frontline screening tool for NF risk stratification in the ED.

**Population Health Research Capsule**

What do we already know about this issue?

The insufficient sensitivity of the Laboratory Risk Indicator for Necrotizing Fasciitis (LRINEC) score makes it an inadequate tool to safely “rule out” necrotizing fasciitis (NF) in the ED.

What was the research question?

To evaluate the predictive ability of the LRINEC score for NF risk stratification in confirmed cellulitis and NF cases.

What was the major finding of the study?

LRINEC score showed high false positive rates in confirmed cellulitis cases and high false negative rates in confirmed NF cases.

How does this improve population health?

NF is a rare disease and the consequences of delayed recognition may be catastrophic. At present, use of LRINEC score cannot be supported as an adequate means to safely exclude NF in the ED setting.

## METHODS

We conducted a 10-year retrospective chart review in the ED that included patients seen at Arrowhead Regional Medical Center (ARMC) from January 1, 2005, to December 31, 2015. ARMC is a 456-bed acute care teaching facility and the only American College of Surgeons-certified Level II trauma center in San Bernardino County, California. 8 The ED at ARMC is the second busiest in the state of California with more than 116,000 annual visits. 12 The institutional review board at ARMC approved this study.

Patients who were ≥18 years old seen at ARMC between January 2005 and December 2015 were assessed for inclusion in this study. We identified patients with cellulitis as the primary or additional diagnosis via ICD-9 discharge diagnosis. Diagnoses of cellulitis were made by EPs following clinical assessment and laboratory findings obtained while in the ED. Exclusion criteria for cellulitis cases included all cases of cellulitis with abscesses that were managed by incision and drainage in the ED, and those patients missing any of the six clinical measures necessary to calculate the LRINEC score (c-reactive protein [CRP], total white blood cell count [WBC], hemoglobin, sodium, creatinine, and glucose).

Patients with a diagnosis of NF were identified via ICD-9 discharge diagnosis. A diagnosis of NF was confirmed through a chart review identifying patients with NF as the primary diagnosis or additional diagnosis, surgical reports that clearly indicated the presence of necrosis in the fascia and subcutaneous tissue, or pathology reports that noted fascial necrosis. Exclusion criteria for NF cases were those directly admitted to the hospital without involvement of the ED, hospital-acquired infections, and transferred patients with prior diagnosis.

Two groups were formed following data collection: cellulitis group (no NF) and NF group. We calculated a LRINEC score ranging from 0–13 for each patient in both groups; LRINEC scores ≥8 fell into the high-risk category, LRINEC scores of 6 or 7 fell into the moderate-risk category, and LRINEC scores ≤5 were considered low risk. 6 All six clinical measures (CRP, total WBC, hemoglobin, sodium, creatinine, and glucose) must have been ordered in the ED for LRINEC calculation to be valid. CRP values for the LRINEC score were either 0 for a negative CRP measurement or 4 for a positive CRP measurement. In cases where a patient was missing a CRP value, a LRINEC score of 0 or 1 without a CRP value would have placed the patient in the “low risk” group per the original guidelines, 6 and a LRINEC score of 8 or greater without a CRP value would have placed the patient in the “high risk” group per the original guidelines. 6 Patients missing CRP values were added to these respective groups.

Since the initial number of patients for the cellulitis group outnumbered those in the NF group, cellulitis patients were randomly selected from only the first week of each month during the study period. Additional variables collected in the cellulitis group were the status of comorbidities, including diabetes.

The primary objective was the predictive ability of the LRINEC decision instrument in patients with a confirmed discharge diagnosis of cellulitis. The secondary objective was the predictive ability of the LRINEC score in patients with a confirmed diagnosis of NF. The impact of comorbidities, including diabetes, on the screening value to the LRINEC score was further assessed in patients with a confirmed discharge diagnosis of cellulitis. Other analyzed factors included each individual value of the LRINEC criteria (CRP, total WBC, hemoglobin, sodium, creatinine, and glucose). We also reviewed and analyzed patients’ demographic data, duration of hospitalization, etiology, underlying systemic disease, bacteriologic and radiologic studies, complications, and treatment outcome.

Residents familiar with study protocol gathered data via retrospective chart review of identified cellulitis and NF cases. Data abstractors had knowledge of the patient’s diagnosis (cellulitis or NF) and were instructed to collect raw data. Data abstractors did not calculate LRINEC scores. All abstracted data were entered into an Excel database. An attending physician was available for consultation/clarification if there were any problems. LRINEC score calculation and classification (low-, moderate-, and high-risk) for each patient were undertaken by the biostatistician.

We conducted all statistical analyses using the SAS software for Windows version 9.3 (Cary, NC). Descriptive statistics were presented as means and standard deviations for continuous variables, along with frequencies and proportions for categorical variables. An independent t-test was conducted to compare the clinical measures between the cellulitis and NF patients. We conducted a Chi-square test to identify the association between the three LRINEC score groups (low, moderate, and high risk) and NF status. Fisher’s exact test was conducted if the expected cell count in each cell was <5. We performed a subgroup analysis to assess the discrimination ability of LRINEC score between diabetes and non-diabetes groups. All statistical analyses were two-sided. P-value<0.05 was statistically significant.

## RESULTS

A total of 3,000 patients were randomly selected from more than 30,000 patients for inclusion in the cellulitis group. We chose 948 patients with CRP values for the cellulitis group. Further breakdown noted 474 diabetes and 474 non-diabetes patients within the cellulitis group. We identified 135 patients for inclusion in the NF group. CRP values were available for 22 (16.3%) patients and we calculated the corresponding LRINEC scores. Furthermore, among the 113 patients without CPR values, six had LRINEC scores ≥ 8 without CRP value, and 19 had LRINEC scores ≤ 1 without CRP value. A total of 47 (the sum of 22, 6 and 19) patients were classified into “low risk,” “moderate risk,” and “high risk” based on LRINEC score.

Table 1 presents the LRINEC scores for the cellulitis group and NF group separately. Based on the LRINEC score risk stratification, among the cellulitis group, 89.2% (n=846 of 948) of the patients were considered as low risk (score ≤5), 6.5% (n=62 of 948) as moderate risk, and 4.2% (n=40 of 948) as high risk for NF. In sum, 10.7% (102 of 948) were misclassified as “at risk” for NF despite a confirmed diagnosis of cellulitis. Among the NF group, 63.8% (n=30 of 47) of the patients were considered as low risk (score ≤5) for NF, 2.1% (n=1 of 47) as moderate risk, and 34% (n=16 of 47) as high risk.

Additionally, we conducted a subgroup analysis of the NF group to identify the discrimination ability of LRINEC between diabetes and non-diabetes patients (Table 2). For the diabetes subgroup with a diagnosis of NF, 43.8% (n=7 of 16) were misclassified as low risk for NF based on LRINEC score. The misclassification rate was more pronounced in the non-diabetes group, with 74.2% (n=23 of 31) misclassified as low risk for NF based on LRINEC score.

For the diabetes subgroup with a diagnosis of cellulitis, 5.5% (n=12 of 474) were misclassified as moderate and 2.5% (n=12 of 474) were misclassified as high risk for NF based on LRINEC score. The misclassification rate was more pronounced in the non-diabetes group with 7.6% (n=36 of 474) misclassified as moderate risk and 5.9% (n=28 of 474) misclassified as high risk for NF based on LRINEC score.

In comparing laboratory values between the cellulitis and NF groups, we found statistically significant differences between the WBC (p<0.0001), serum sodium level (p<0.0001), creatinine level (p<0.0001), glucose level (p<0.0001) and CRP level (p=0.0035). However, no difference was detected in hemoglobin levels between the cellulitis and NF group (p=0.149). When stratifying based on diabetes status, WBC, sodium, creatinine, glucose, and CRP were significantly different between the cellulitis and NF groups, while hemoglobin was not significantly different.

## DISCUSSION

The current study suggests that the LRINEC score may not be an accurate tool for NF risk stratification and differentiation between cellulitis and NF in the ED setting. Among patients with confirmed diagnoses of cellulitis, 10.7% were categorized as moderate to high risk for NF based on the LRINEC score. The high incidence of false positives adds a new dimension to investigations seeking to assess the validity of the LRINEC score. To our knowledge, no study has been conducted to evaluate the efficacy of the LRINEC decision instrument against a large sample of patients with a confirmed diagnosis of cellulitis.

Additionally, among patients with confirmed diagnoses of NF, 63.8% were categorized as low risk for NF based on the LRINEC score. Based on the initial LRINEC validation study by Wong et al., this decision instrument carries a positive predictive value of 92% and negative predictive value of 96%.^6^ However, a subsequent retrospective analysis of the LRINEC score noted a sensitivity of only 77% when assessing against confirmed cases of NF.^9^ In addition, multiple other studies reported inadequate sensitivity of the LRINEC score to rule out NF in cases of confirmed NF.^8^,^9^,^13^

Based on the results of the current study, using the LRINEC score for NF risk stratification in cases of confirmed cellulitis at our institution could have resulted in a misleading differential diagnosis, leading to a more rigorous clinical workup and treatment protocol that are normally associated with NF. The possibility of invasive intervention would have been higher, further exacerbating the emotional, physical, and financial burdens for these patients. Over 30,000 patients with a diagnosis of cellulitis were originally assessed for inclusion in this study. If the LRINEC score had been used in isolation to direct the clinical management of these patients, 10.7%, or more than 3,000 individuals, would have been subjected to inappropriate management.

Additionally, the current study assessed the LRINEC decision instrument misclassification rate among diabetes patients versus non-diabetes patients. The misclassification rate was 8% and 13.5% among diabetic and non-diabetic patients among the cellulitis group, respectively. Similarly, among the NF group, the misclassification rate was 43.8% among diabetic patients and 74.2% among non-diabetic patients. It appears that the LRINEC scoring tool more accurately assessed NF risk stratification among diabetic patients in comparison to the non-diabetic patients. This finding is consistent with Burner et al., who reported a better discrimination ability of the LRINEC score for NF cases among the diabetic population.^9^

## LIMITATIONS

This study was limited by the inability to calculate a complete LRINEC score in the majority of patients with suspected NF due to a lack of CRP measured in the ED. The non-specific nature of CRP as a marker of systemic inflammation in numerous disease processes reduces its relevance as a routinely ordered test.^14^,^15^ Similar limitations were reported from several other studies attempting to validate the LRINEC score.^9^,^14^ In the current study, a LRINEC score could only be calculated in 22 patients with a confirmed NF diagnosis as they were the only cases with all six components measured in the ED (CRP, total WBC, hemoglobin, sodium, creatinine, and glucose). To increase sample size and strengthen the generalizability of findings in the current study, we further included 25 additional patients who were in the low-risk or high-risk group even without the CRP values.

Another limitation is the small sample size. However, the majority of present literature consists of small sample size studies and case reports. Given that NF is a rare disease process, generating a large sample size was a significant obstacle.

## CONCLUSION

In the ED setting, the LRINEC score may not be an accurate tool to determine NF risk stratification or to differentiate between cellulitis and NF. This decision tool demonstrated a high false positive rate when classifying NF risk stratification in confirmed cases of cellulitis and a high false negative rate in cases of confirmed NF. Emergency physicians should be cognizant of the limitations of the LRINEC score and continue to carry a high index of suspicion in patients who present with pain out of proportion, signs of skin necrosis, and subcutaneous gas on imaging studies.

## Figures and Tables

**Table 1:** Laboratory risk indicator for necrotizing fasciitis (LRINEC) score for necrotizing fasciitis and cellulitis.

	NF (n=135)	Cellulitis (n=948)	p-value
LRINEC Groups			<.0001
High risk: LRINEC ≥8	16 (34%)	40 (4.2%)	
Moderate risk: LRINEC 6 and 7	1 (2.1%)	62 (6.5%)	
Low risk: LRINEC ≤5	30 (63.8%)	846 (89.2%)	
	Missing = 88		
WBC (*1000 per mm3)	18.32 ± 9.16	9.52 ± 5.39	<.0001
Hemoglobin (g/dL)	12.87 ± 2.36	12.57 ± 2.21	0.149
Sodium (mmol/L)	131.77 ± 6.05	137.69 ± 3.84	<.0001
Creatinine (umol/L)	160.66 ± 155.05	91.7 ± 93.72	<.0001
Glucose (mmol/L)	13.61 ± 11.13	9.27 ± 5.41	<.0001
CRP (mg/dL)	178.06 ± 165.42	61.68 ± 92.09	0.0035
Age, years	47.31 ± 13.05	50.33 ± 13.78	0.0166

*LRINEC*, laboratory risk indicator for necrotizing fasciitis; *NF*, necrotizing fasciitis; *WBC*, white blood cell; *CRP*, c-reactive protein.

All percentages may not add up to 100% by each column due to rounding.

To change the creatinine from umol/L to mg/dl, use the formula mg/dl=88.4*umol/L.

To change glucose from mmol/L to mg/dL, use the formula mg/dL=0.055 mmol/L.

To change CPR from mg/dl to mg/L, use the formula mg/L=0.1* mg/dL.

**Table 2:** Laboratory risk indicatory for necrotizing fasciitis (LRINEC) score for necrotizing fasciitis and cellulitis by diabetes status.

	Diabetes subgroup			Non-diabetes subgroup		
		
	NF (n=16)	Cellulitis (n=474)	p-value	NF (n=31)	Cellulitis (n=474)	p-value
LRINEC groups			0.0012			<.0001
High risk: LRINEC ≥8	9 (56.3%)	12 (2.5%)		7 (22.6%)	28 (5.9%)	
Moderate risk: LRINEC 6 and 7	0 (0%)	26 (5.5%)		1 (3.2%)	36 (7.6%)	
Low risk: LRINEC ≤5	7 (43.8%)	436 (92%)		23 (74.2%)	410 (86.5%)	
	Missing = 41			Missing = 47		
WBC (*1000 per mm3)	18.13 ± 10.57	9.76 ± 4.94	<.0001	18.22 ± 6.33	9.27 ± 5.8	<.0001
Hemoglobin (g/dL)	13.11 ± 2.33	12.93 ± 2.03	0.4827	12.38 ± 2.35	12.21 ± 2.32	0.6003
Sodium (mmol/L)	133.08 ± 5.35	138.04 ± 3.47	<.0001	129.65 ± 6.47	137.33 ± 4.15	<.0001
Creatinine (umol/L)	159.27 ± 158.23	77.04 ± 77.23	<.0001	159.15 ± 158.38	106.36 ± 105.78	0.0225
Glucose (mmol/L)	7.54 ± 3.76	6.42 ± 2.15	0.013	22.2 ± 12.62	12.12 ± 6.14	<.0001
CRP (mg/dL)	167.64 ± 153.64	62.6 ± 87.92	0.0194	200.4 ± 199.61	60.75 ± 96.17	0.0002
Age, years	45.93 ± 14.16	46.46 ± 14.66	0.7725	49.5 ± 10.53	54.21 ± 11.61	0.0053

*LRINEC*, laboratory risk indicator for necrotizing fasciitis; *NF*, necrotizing Fasciitis; *WBC*, white blood cell; *CRP*, c-reactive protein.

To change the creatinine from umol/L to mg/dL, use the formula mg/dL=88.4*umol/L.

To change glucose from mmol/l to mg/dL, use the formula mg/dL=0.055 mmol/L.

To change CPR from mg/dL to mg/L, use the formula mg/L=0.1* mg/dL.
